# A training programme for medical students in providing spiritual care to people with advanced diseases and their loved ones: A case study from the Collegium Medicum in Bydgoszcz, Nicolaus Copernicus University in Toruń, Poland

**DOI:** 10.3389/fcvm.2022.909959

**Published:** 2022-09-29

**Authors:** Małgorzata Fopka-Kowalczyk, Richard Groves, Philip Larkin, Małgorzata Krajnik

**Affiliations:** ^1^Department of Philosophy and Social Sciences, Nicolaus Copernicus University in Toruń, Torun, Poland; ^2^Sacred Art of Living Center, Bend, OR, United States; ^3^Palliative and Supportive Care Service, Chair of Palliative Care Nursing, Lausanne University Hospital and University of Lausanne, Lausanne, Switzerland; ^4^Department of Palliative Care, Nicolaus Copernicus University in Toruń, Collegium Medicum in Bydgoszcz, Bydgoszcz, Poland

**Keywords:** spiritual care, spiritual curriculum, education on spirituality in medicine, medical students, spiritual needs of people with advanced illness

## Abstract

**Purpose:**

This article presents the first programme on spiritual care particularly for people with advanced life-limiting illness including heart failure, lung disease or cancer for medical students in Poland implemented at the Collegium Medicum in Bydgoszcz of the Nicolaus Copernicus University in Toruń.

**Methods and materials:**

Several steps were identified for the development of the first programme on spirituality for medical students at the Collegium Medicum in Bydgoszcz including preliminary work on the content of the programme, agreement on key concepts, terms, and definitions; consultations with teachers and review of the literature.

**Results:**

The first Polish spiritual curriculum for medical students was implemented. The spirituality curriculum will potentially contribute to better care for the people with advanced illnesses such as heart failure, chronic lung disease or cancer and improve the quality of relationships between professionals and patients.

**Conclusion:**

The article presents the content of the program, the expected learning objectives and ascribed teaching methods, along with the preliminary evaluation made by students.

## Introduction

Caring for people with advanced illnesses such as heart failure, chronic lung disease or cancer is a significant challenge for healthcare professionals. Diagnosing patients' needs, preparing treatment plans, and providing support require a deep engagement. The resolution of complex clinical situations may be predicated on the relationship that develops between a professional and a person in need of help. That relationship between a healthcare professional and a patient is particularly challenged through the situation of illness, suffering, or death, which requires intimate knowledge regarding the life history. By promoting an holistic approach to patient care, where one is focused on whole-person caregiving as opposed to a single organ that is failing, the breadth of knowledge of medicine and medical protocols may prove to be insufficient (1). The wider impact of this life-changing event also needs to be considered. Medicine is a field in which the influence of personal, emotional, and spiritual aspects on the course of illness and/or patients' recovery and emotional balance is prominent. Enhanced spiritual care has a favorable effect on survival (2), demonstrates better coping with disease (3), patient satisfaction with treatment and care (4), compliance with treatment (5), greater well—being and quality of life (6–10) as well as reduced anxiety and depression (11–15). Patients are also more able to cope with their disease and have more positive attitude despite their difficult health situation (3, 15). The relationship with quality of life, coping with the disease, and spiritual support received confirm that spirituality is an essential part of human life and patient care (1, 16, 17). Looking at patients holistically sets new standards of treatment, care and support, necessitating healthcare professionals to learn interpersonal skills which include the ability to care for patients' spiritual needs and understand, where possible, their existential quest (18). Such an approach enforces changes in medical curricula to ensure that students receive substantive learning in relation to whole person care. This article presents the first such programme as a curriculum for medical students in Poland. It discusses the development of the “Spirituality in Medicine” programme and its successful implementation at the Collegium Medicum in Bydgoszcz of the Nicolaus Copernicus University in Toruń.

## Methods

**Figure 1:**
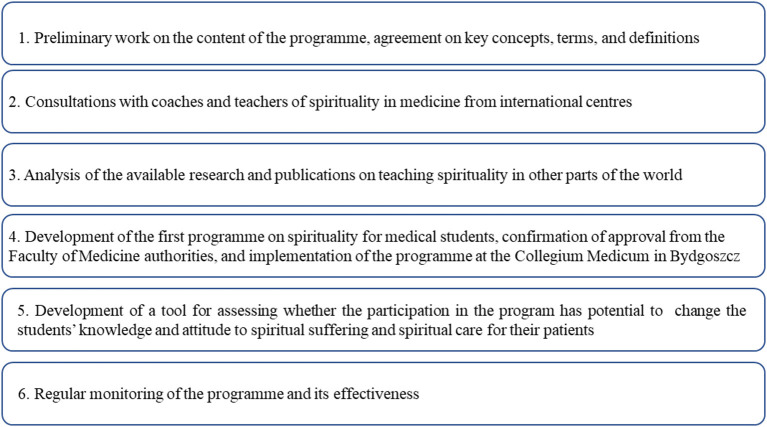
Key areas of development of the “Spirituality in Medicine” programme.

The “Spirituality in Medicine” Programme supported by the Polish Association for Spiritual Care in Medicine (PASCiM), was launched in 2017 and several focus areas were identified (Figure 1).

### Preliminary work on the content of the programme, agreement on key concepts, terms, and definitions

A curriculum capable of meeting the needs of professionals should ideally be founded on a well-defined concept. It was therefore necessary to develop a common definition of spirituality, covering the sphere of human religious experience and practice, existential search and other significant values in human life. Adopting such a construct offered medical students an opportunity to examine the deepest layers of what it means to be human, areas which can provide strength but may also cause a personal struggle. Through this approach it was proposed that it would be easier for them to realize that their patients can suffer not only physically but also spiritually; consequently they are in need of spiritual care and support, which should be an integral part of the treatment process (19).

Two definitions of spirituality were used for the development of the assumptions and plan for the programme. The first was the definition proposed by the European Association for Palliative Care (EAPC) Task Force in 2011 (revised in 2020), who consider “spirituality is the dynamic dimension of human life that relates to the way persons (individual and community) experience, express and/or seek meaning, purpose and transcendence, and the way they connect to the moment, to self, to others, to nature, to the significant and/or the sacred” (revised definition from 2011) (20). The second definition was developed by PASCiM which defines spirituality as a dimension of human life that relates to transcendence and other existentially important values (21, 22). Based on the EAPC approach to spirituality, PASCiM similarly recognizes three dimensions of spiritual experience which include: religiousness of a person, especially his/her relationship with God, personal beliefs, and religious practices, as well as community interaction; existential quest, especially with regard to the meaning of life, suffering, and death, issues of own dignity, personhood, a sense of individual freedom and responsibility, hope and despair, reconciliation and forgiveness, love and joy; values by which a person lives, especially with regard to oneself and others, work, nature, art and culture, ethical and moral choices, and life itself (20–22). These definitions provided the basis for planning the program.

### Consultations with international teachers of spirituality in medicine

Another factor that made a significant contribution to the programme's final shape was extensive consultations with international experts in the teaching of spirituality in medicine. Contribution to the development of the curriculum included visits to the University, engagement in workshops and seminars, teaching of medical students and evaluation of content as a basis for the development of a new curriculum. Such exposure to current international examples of spiritual care education enabled a better understanding of which elements and tools were potentially transferable to the Polish context and ensured parity with current international standards.

### Analysis of the available research and publications on teaching spirituality

A review of existing international programmes was conducted (23–27). This included an in-depth analysis of the course curricula and teaching methods to underpin the proposed programme. Consequently, ideas were chosen that were not only in line with the adopted definitions but also in compliance with the highest available global standards of spirituality teaching.

### Development and implementation of the first programme on spirituality for medical students

The proposed programme as a compulsory course for medical students was approved by the Faculty of Medicine of the Collegium Medicum in Bydgoszcz, Nicolaus Copernicus University in Toruń, and introduced for the first time during the 2018/2019 academic year. It envisaged work with students throughout the subsequent years of studies (i.e., from 2^nd^ to 5^th^ year, a total of 48 teaching hours) (Table 1). In the years 2019/2020 and 2020/2021, the entire programme was implemented over a course of 22 teaching hours for the second-year students. However, from the academic year 2021/2022 it is now taught during the 2^nd^ and 5^th^ years of medical studies (the latter as a part of palliative medicine module) reflecting a change in the general strategy of University. As a result, these developments have created the unique opportunity to monitor and compare the efficacy of different approaches to teaching spirituality to medical students regarding the question if it is better to teach spirituality every year or just at the beginning and at the end of medical studies.

**Table 1:** An outline of the obligatory programme of education in spirituality for medical students at the Collegium Medicum in Bydgoszcz, Nicolaus Copernicus University in Toruń.

**Form**	**2^nd^ year (*n* = 189)**	**3^rd^ year (*n* = 177)**	**4^th^ year (*n* = 177)**	**5^th^ year (*n* = 175)**
**Program implemented in 2018/2019**				
	Introduction to spirituality in medicine. Basic spiritual care provided by doctors. Specialist spiritual care. Total pain, spiritual pain and suffering. Compassion in clinical practice. Communication and own pathway to be a doctor.	Mindful presence or spirituality in clinical practice. Diagnosing spiritual needs. Non–violent communication.	Helping family to say goodbye to loved one who is dying alone in hospital from COVID-19. Spiritual care for the patient with COVID-19. Spiritual care from the perspective of psychologist. Cooperation with the chaplain. Dignity Therapy. How doctor can support his/her patient.	Developing hospital program on spiritual care. Helping find meaning. Communication about spirituality.
Lectures	4	4	-	-
Seminars	4	4	8	4
Workshops	4	4	4	8
**Program implemented in 2019/2020 and 2020/2021**				
Form	2^nd^ year (in 2019/2020: *n* = 266; in 2020/2021: *n* = 263)			
	Introduction to spirituality in medicine. Basic spiritual care provided by doctors and specialist spiritual care. Hospital programs dedicated to spiritual care. Diagnosing spiritual needs. Dealing with hope. Compassion in clinical practice or spiritual care in clinical practice. Clinical case—spiritual care in psychiatry. Dignity Therapy. Non–violent communication.			
Lectures	10			
Seminars	6			
Workshops	6			
**Program implemented since 2021/2022**				
Form	2^nd^ year(as “Spiritual care”) (*n* = 216)		5^th^ year (spiritual care as the part of palliative medicine module)	
	Introduction to spirituality in medicine. Basic spiritual care provided by doctors. Hospital programs dedicated to spiritual care. Healing in medicine. Diagnosing spiritual needs. Compassion in clinical practice. Communication about spirituality. Cooperation with healthcare chaplain.		Interventions for spiritual distress: dignity therapy and helping find meaning. Communication about spirituality. Mindful presence	
Lectures	4		-	
Seminars	-		6	
Workshops	6		6	

n, number of students participating in the programme.

### Development of a tool for assessing whether the participation in the program changes the students' knowledge and attitude to spiritual suffering and spiritual care for their patients

It was crucial to assess not only the knowledge of spirituality as acquired by medical students, but also the impact of teaching spirituality on the improvement of their skills and sensitivity in this area, therefore enhancing the students' competences as future doctors. Students were asked to fill in a questionnaire before and after their participation in the programme on spirituality. This structured and standardized psychometric tool Spiritual Support Scale (SpSup) Scale have been described elsewhere (28).

### Regular monitoring of the program and its effectiveness

In addition to the SpSup Scale, two other tools have been applied to evaluate the classes:

1. A questionnaire for the evaluation of the program at the end of their course, consisting of three open questions (what was the most useful? what was the least useful? what do I propose to change or include?).
2. Qualitative interviews conducted with a sample of students' of each class by open invitation. Interviews were conducted at the end of the course, were anonymous and voluntary. Participants were asked to consider the following five questions:
  - What do you think about the spirituality classes in which you participated?
  - What is the meaning of the spirituality classes to you as a future doctor as well as in your own life?
  - What advantages and disadvantages could you see in the spirituality program (what subjects were not discussed enough and which ones should be excluded)?
  - In what way has the spirituality curriculum changed your view on spirituality?
  - Is this sphere important to you in own life and your work with people who are ill? In what way?

The interviews lasted about 30–45 min and were recorded with the agreement of the participants.

## Results

The overarching aim of our “Spirituality in Medicine” program was to provide medical students with knowledge about spirituality (as defined above) and improve their competences in this area. The subjects taught during the classes as well as the expected learning outcomes correspond to the following objectives:

- To learn about spirituality as defined in the program and how to refer this knowledge to one's own experiences, beliefs, and values;
- To learn about compassion in the context of spiritual care as a key aspect of interpersonal skills;
- To gain competence in diagnosing patients' spiritual needs and suffering;
- To learn how to provide help and spiritual support corresponding to the spiritual needs reported by patients (e.g., non-aggressive communication, interpersonal communication, mindful listening);
- To learn the necessary methods, techniques, and research tools to diagnose patients' spiritual needs and suffering;
- To learn about possible therapeutic methods that can be used when providing spiritual care to patients;
- To be able to cooperate—as a physician—with a chaplain;
- To learn how to help patients examine their lives, find a meaning and leave a spiritual legacy (e.g., elements of dignity therapy) (29).

Table 1 provides a general outline of the program, highlighting in particular:

- The academic year for which a given module was intended;
- Topics to be discussed during the classes;
- The expected learning outcomes.

In our classes, all students of each year participated in mandatory lectures and workshops (Table 1).

Regardless of the number of teaching hours allocated to this subject, we wanted students to have the opportunity to gain similar knowledge and skills in core aspects of spiritual care and spirituality. The amount of material covered varied depending on the time available for the respective thematic block.

In order to achieve the intended aims and objectives and ensure expected learning outcomes, a number of teaching methods were proposed and have since been implemented during the classes. The most important of these are thematic lectures, as well as seminars and workshops.

Other techniques employed during the classes were as follows.

• **Excerpts from films, case studies, stories** - Using various aids related to topics covered during the classes to apply the newly acquired knowledge and skills to concrete examples.
• **Role play, psychodrama** - To practice new skills.
• **Students sharing their own experiences** - In order to analyse their own emotions and experiences and thus better understand themselves while also highlighting possible reactions as well as the spiritual needs, suffering, and experiences of patients.
• **Interactive discussion** - Addressing problems faced during the classes after watching a film or studying a case.
• **Students' own work based on case study presentation** - Writing papers focused on the specific case study, including proposed spiritual interventions and techniques to be implemented.
• **Students sharing their experiences from practice** - Based on direct contacts with patient.

The initial analyses of the interviews show that our classes were initially approached with reserve and reluctance. At the beginning, some students thought that the main aim of the programme was to “convert” them or “discuss religion”. They had doubts about whether it really should be part of “a doctor's work”. However, as the course progressed, they started to appreciate the variety of topics covered. With time they changed their minds and evaluated the course positively. Interviewees clearly expressed their appreciation for the newly acquired skills. They also found valuable that the classes presented them with different methods of diagnosing spiritual needs or suffering, conducting an intervention, and providing support.

The interviews with students participating in the classes indicate that students particularly enjoyed working on case studies and discussions based on films that dealt with the topics taught during the classes. Indeed, there was a belief that most of the issues covered during the course would prove useful in their future work, particularly in the case of students who plan to work with chronic disease patients.

Although we invited all students, who participated in the classes, only 5 agreed to be interviewed. However, qualitative research samples are usually small and sufficient data saturation was achieved with this number of interviews (30).

## Discussion

This is the first compulsory spiritual care programme offered in a medical school in Poland. Teaching future physicians how to engage with spiritual care is essential for the development of whole person care medicine (19, 23, 26). Physicians are not only “technically-competent” specialists in the specific branches of medicine, but compassionate persons accompanying other persons who are suffering, searching for the meaning, real hope, forgiveness, or closeness to God and/or other people (23).

How physicians builds the relationship with patients depends on a blend of professional knowledge, communication, compassionate presence and understanding of the experience of illness and suffering. To enable this, medical curricula should be directed toward improvement of skills and competences needed to address spiritual care (31), the aim of this first spiritual care in medicine program in Poland.

The aims and objectives of our program cited earlier are similar to those defined for the educational programs introduced at medical universities in United States, South America, United Kingdom and some other European countries (24–27, 31–39). These different spirituality curricula are generally focused on improving the student mindfulness, compassion and empathy, careful listening and communication on what is integral to the person, what he/she believes in, and hopes for. These also frame the starting point for the Polish curriculum (24, 26, 40).

Programs concentrating on improving the ability to perform spiritual care are usually based on case discussions, real patient history taking or self-reflective journaling (27). These complement the structured clinical knowledge achieved in a medical degree and are an important component of holistic mastery in clinical medicine. Thus, education on spirituality should be at least partially included during the clinical years of studying medicine. As we monitor the efficacy of teaching spirituality to medical students, we hope in the near future to assess whether it is better to teach spirituality every year, only at the beginning or just at the beginning and at the end of medical studies.

A recent systematic review of international medical school and residency program curricula that address spiritual care pointed also to some other core content not included in our current curriculum, such as chaplain shadowing, OSCE (Objective Structured Clinical Examination), simulated patients or spirituality dinners (27).

To date, the program on spirituality in medicine as a curriculum is provided only in one medical university in Poland. Future analysis of its effects on students' attitude and practice would help to determine the relevance of such education.

## Conclusions

Working with patients, particularly those with advanced illnesses such as heart failure, chronic lung disease or cancer at the end of life, reveals how patients develop an intrinsic curiosity in their life situation, show interest in their medical and human needs, and seek compassion. Patient wish to derive some meaning to their experience. They seek answers and have a need to talk about that experience, even if difficult or challenging for them.

The proposed programme of teaching spirituality to future doctors is the first educational project of this type in Poland. It is our hope that this new curriculum will equip future doctors to respond sensitively and appropriately to the complex questions raised by illness, frailty and death.

## Data availability statement

The raw data supporting the conclusions of this article will be made available by the authors, without undue reservation.

## Ethics statement

The studies involving human participants were reviewed and approved by the Ethics Committee of the Collegium Medicum in Bydgoszcz of Nicolaus Copernicus University in Toruń, Poland (KB 736/2018). In accordance with the local legislation and institutional requirements, written informed consent was not required for the anonymous survey among students.

## Author contributions

MF-K and MK contributed to the creation and development of the program along to the process of its implementation and monitoring. PL and RG contributed to the preliminary work on the content of the program. MF-K was responsible for the critical analysis of the literature on teaching spirituality in other parts of the world and for drafting the first version of the manuscript. MK was a coordinator of the program in the University. All authors were involved in critical analysis, interpretation of the study results, and preparation of the final version of the manuscript.

## Funding

Some steps of the project were financed by Nicolaus Copernicus University in Torun Internal University Grant No. WN949. In addition Open access funding was paid for by the Faculty of Biology and Medicine, University of Lausanne, Switzerland.

## Conflict of interest

The authors declare that the research was conducted in the absence of any commercial or financial relationships that could be construed as a potential conflict of interest.

## Publisher's note

All claims expressed in this article are solely those of the authors and do not necessarily represent those of their affiliated organizations, or those of the publisher, the editors and the reviewers. Any product that may be evaluated in this article, or claim that may be made by its manufacturer, is not guaranteed or endorsed by the publisher.
